# Predictive Role of Neutrophil-to-Lymphocyte Ratio and Serum Creatinine in the Early Detection of Sepsis-Related Organ Dysfunction

**DOI:** 10.7759/cureus.113217

**Published:** 2026-07-23

**Authors:** Ahmed Jamal Chaudhary, Manahil Mariam, Rafia Hassan, Muhammad Ilyas, Sara Khan, Abdul Wasay Javed, Syed Muhammad Abdullah, Imran Ali Shaikh

**Affiliations:** 1 Internal Medicine, DMC Sinai-Grace Hospital, Detroit, USA; 2 Medicine, Multan Medical & Dental College, Multan, PAK; 3 Medicine, Rawal Institute of Health Sciences, Islamabad, PAK; 4 Internal Medicine, Watim Medical & Dental College, Rawalpindi, PAK; 5 Pathology, CMH Institute of Medical Sciences Bahawalpur, Bahawalpur, PAK; 6 Physiology, M. Islam Medical and Dental College, Gujranwala, PAK; 7 Cardiothoracic Surgery, Tabba Heart Institute, Karachi, PAK; 8 Medicine, Liaquat University of Medical & Health Sciences, Jamshoro, PAK

**Keywords:** biomarkers, neutrophil-to-lymphocyte ratio, organ dysfunction, sepsis, serum creatinine

## Abstract

Background and aim

Sepsis is a major cause of morbidity and mortality worldwide. Early identification of patients at risk of organ dysfunction is important for timely treatment and improved outcomes. Although neutrophil-to-lymphocyte ratio (NLR) and serum creatinine are not specific to sepsis and may be elevated in other inflammatory or renal conditions, they are simple and readily available laboratory markers that may help identify high-risk patients at hospital admission. The aim of this study was to evaluate the predictive role of NLR and serum creatinine in the early detection of sepsis-related organ dysfunction.

Methods

This cross-sectional study was conducted at Mekran Medical College, Turbat, Pakistan, from January 2025 to June 2025. A total of 384 adult patients with sepsis were enrolled through consecutive sampling. Demographic and clinical data were collected at hospital admission. CBCs and serum creatinine levels were measured at admission, and NLR was calculated by dividing the absolute neutrophil count by the absolute lymphocyte count. Receiver operating characteristic (ROC) curve analysis and multivariable logistic regression were performed to evaluate the association and predictive performance of the studied markers.

Results

Among 384 patients, 142 (37%) developed sepsis-related organ dysfunction. Patients with organ dysfunction had significantly higher NLR (12.4 ± 4.6 vs. 5.4 ± 2.2, p < 0.001) and serum creatinine levels (2.11 ± 0.89 vs. 1.09 ± 0.34 mg/dL, p < 0.001). ROC analysis showed that NLR had an area under the curve (AUC) of 0.868, while serum creatinine had an AUC of 0.831. Multivariable logistic regression demonstrated that NLR (adjusted OR = 1.28, 95% CI: 1.20-1.36, p < 0.001) and serum creatinine (adjusted OR = 3.04, 95% CI: 2.06-4.50, p < 0.001) were independent predictors of organ dysfunction, although the wider confidence interval for serum creatinine suggests lower precision of the estimated effect.

Conclusions

Elevated NLR and serum creatinine were significantly associated with sepsis-related organ dysfunction at hospital admission. Both markers may serve as simple, inexpensive, and readily available tools for early risk assessment of septic patients. However, the cross-sectional design limits causal inference, and prospective studies are needed to confirm their predictive value over time.

## Introduction

Sepsis is a serious medical condition caused by an abnormal and uncontrolled response of the body to infection [1]. It can rapidly progress to organ dysfunction, septic shock, and death if not recognized early. According to the Sepsis-3 definition, sepsis is a life-threatening organ dysfunction caused by a dysregulated host response to infection [2]. Early recognition of organ dysfunction remains one of the most important steps in the management of septic patients.

Sepsis remains a major public health challenge in low- and middle-income countries, including those in South Asia, where delayed diagnosis, limited healthcare resources, and high infection rates contribute to poor clinical outcomes. In Pakistan, early recognition of sepsis is often challenging because access to advanced diagnostic tools, including biomarkers such as procalcitonin and IL-6, and timely microbiological investigations may be limited, particularly in resource-constrained hospitals. Consequently, readily available laboratory markers are of considerable clinical value for early risk assessment [3]. Therefore, there is a need for simple, inexpensive, and readily available biomarkers that can help identify patients at risk of organ dysfunction during the early stages of sepsis.

Inflammation plays a central role in the development of sepsis-related organ injury. During infection, neutrophils increase as part of the body’s first immune response, while lymphocyte counts often decrease because of immune dysregulation and cellular apoptosis [4]. The neutrophil-to-lymphocyte ratio (NLR) combines these two changes into a single marker. Because it can be calculated from a routine CBC, NLR has gained attention as a practical indicator of systemic inflammation [5]. Recent systematic reviews and meta-analyses have reported that elevated NLR is associated with greater disease severity, poor prognosis, and increased mortality among septic patients [6,7].

Kidney dysfunction is one of the most common complications of sepsis. Reduced tissue perfusion, inflammatory injury, and microvascular disturbances may impair renal function during the early phase of the disease [8]. Serum creatinine is an inexpensive and routinely performed laboratory test that is available in most secondary and tertiary healthcare facilities and in many primary healthcare settings. Its widespread use makes it a practical marker for assessing kidney dysfunction in patients with suspected sepsis, particularly where access to advanced biomarkers is limited [9]. An increase in serum creatinine may indicate early organ dysfunction and help clinicians identify patients who require closer monitoring and aggressive treatment [10].

Although several international studies have evaluated NLR and serum creatinine individually, evidence regarding their combined role in predicting sepsis-related organ dysfunction remains limited in Pakistan. This study was therefore conducted to address this important local evidence gap. However, as the findings are derived from a single-center population, further multicenter studies are needed to confirm their generalizability across different healthcare settings and populations. The present study was conducted to assess the predictive role of NLR and serum creatinine in the early detection of sepsis-related organ dysfunction among patients presenting to a tertiary care hospital in Pakistan.

## Materials and methods

This is cross-sectional research, and it was conducted at M. Islam Medical and Dental College, Gujranwala, Pakistan, from January 2025 to June 2025. The study was approved by the Institutional Ethical Review Board of M. Islam Medical and Dental College (approval 492/24, dated November 15, 2024). Informed consent was obtained in writing from all the patients to include them in this research.

The sample size was calculated using an expected prevalence of 50%, a 95% confidence level, and a 5% margin of error. A prevalence of 50% was selected because reliable local estimates of sepsis-related organ dysfunction were unavailable. This conservative assumption provides the maximum sample size required when the expected prevalence is uncertain. The calculated sample size was 384 participants [11].

Adult patients with suspected or confirmed infection were screened at hospital admission. Initial bedside assessment was performed using the quick Sequential Organ Failure Assessment (qSOFA) score, which included a respiratory rate of at least 22 breaths per minute, altered mental status, and systolic blood pressure of 100 mmHg or less [12]. Patients with a qSOFA score of at least 2 underwent further clinical and laboratory assessment. Sepsis-related organ dysfunction was assessed using the Sequential Organ Failure Assessment (SOFA) score [13].

Demographic data and clinical information, including age, gender, comorbidities, vital signs, and laboratory findings, were collected from patient interviews and hospital records using an author-developed data collection pro forma (Appendix A). Venous blood samples were obtained at admission. CBCs were performed using an automated hematology analyzer. The NLR was calculated by dividing the absolute neutrophil count by the absolute lymphocyte count. Serum creatinine was measured using a standard enzymatic method in the hospital laboratory. Sepsis-related organ dysfunction was identified based on the presence of acute organ impairment documented by the treating physician and supported by laboratory and clinical findings.

Sepsis-related organ dysfunction was defined as an acute increase of at least 2 points in the SOFA score from baseline. The SOFA score was calculated using respiratory, cardiovascular, hepatic, coagulation, neurological, and renal parameters recorded at hospital admission. In patients without known pre-existing organ dysfunction, the baseline SOFA score was assumed to be zero. This standardized definition was used instead of relying solely on the treating physician’s clinical documentation.

Data were analyzed using IBM SPSS Statistics for Windows, version 26.0 (released 2018; IBM Corp., Armonk, NY, USA). Continuous variables were presented as mean ± SD, while categorical variables were reported as frequency and percentage. Independent-samples t-test and chi-square test were used for group comparisons where appropriate. Receiver operating characteristic (ROC) curve analysis was performed to evaluate the predictive performance of NLR and serum creatinine for early detection of sepsis-related organ dysfunction. Multivariable logistic regression analysis was used to identify independent predictors after adjustment for potential confounders. A p-value of less than 0.05 was considered statistically significant.

## Results

A total of 384 patients with sepsis were enrolled in the study. Among them, 142 (37%) developed sepsis-related organ dysfunction, while 242 (63%) did not. The mean age of the study population was 55.2 ± 16.1 years, and 229 (59.6%) participants were male.

Patients with organ dysfunction were significantly older than those without organ dysfunction (61.8 ± 15.9 vs. 51.3 ± 15.2 years, p < 0.001). Diabetes mellitus, hypertension, and chronic kidney disease were also more common among patients with organ dysfunction (all p < 0.05) (Table 1).

**Table 1:** Baseline characteristics of study participants according to organ dysfunction status Values are shown as mean ± SD and frequency (%). Independent-samples t-test was used for continuous variables and chi-square test for categorical variables.

Variable	No organ dysfunction (n = 242)	Organ dysfunction (n = 142)	p-value
Age (years), mean ± SD	51.3 ± 15.2	61.8 ± 15.9	<0.001
Male gender, n (%)	137 (56.6)	92 (64.8)	0.113
Diabetes mellitus, n (%)	84 (34.7)	79 (55.6)	<0.001
Hypertension, n (%)	102 (42.1)	79 (55.6)	0.011
Chronic kidney disease, n (%)	27 (11.2)	36 (25.4)	<0.001
Respiratory infection, n (%)	96 (39.7)	63 (44.4)	0.366
Urinary tract infection, n (%)	77 (31.8)	35 (24.6)	0.136
Abdominal infection, n (%)	46 (19.0)	31 (21.8)	0.510
Other infections, n (%)	23 (9.5)	13 (9.2)	0.906

Laboratory findings are summarized in Table 2. Patients with organ dysfunction had significantly higher neutrophil counts, white blood cell counts, NLR values, and serum creatinine levels, while lymphocyte counts were significantly lower (all p < 0.001).

**Table 2:** Laboratory parameters of study participants Values are presented as mean ± SD. Independent sample t-test was used for comparison between groups. NLR: neutrophil-to-lymphocyte ratio

Variable	No organ dysfunction (n = 242)	Organ dysfunction (n = 142)	p-value
Neutrophil count (× 10⁹/L)	8.8 ± 2.6	12.9 ± 3.5	<0.001
Lymphocyte count (× 10⁹/L)	1.84 ± 0.63	1.07 ± 0.43	<0.001
NLR	5.4 ± 2.2	12.4 ± 4.6	<0.001
Serum creatinine (mg/dL)	1.09 ± 0.34	2.11 ± 0.89	<0.001
White blood cell count (× 10⁹/L)	12.1 ± 3.8	15.8 ± 4.9	<0.001

**Figure 1:**
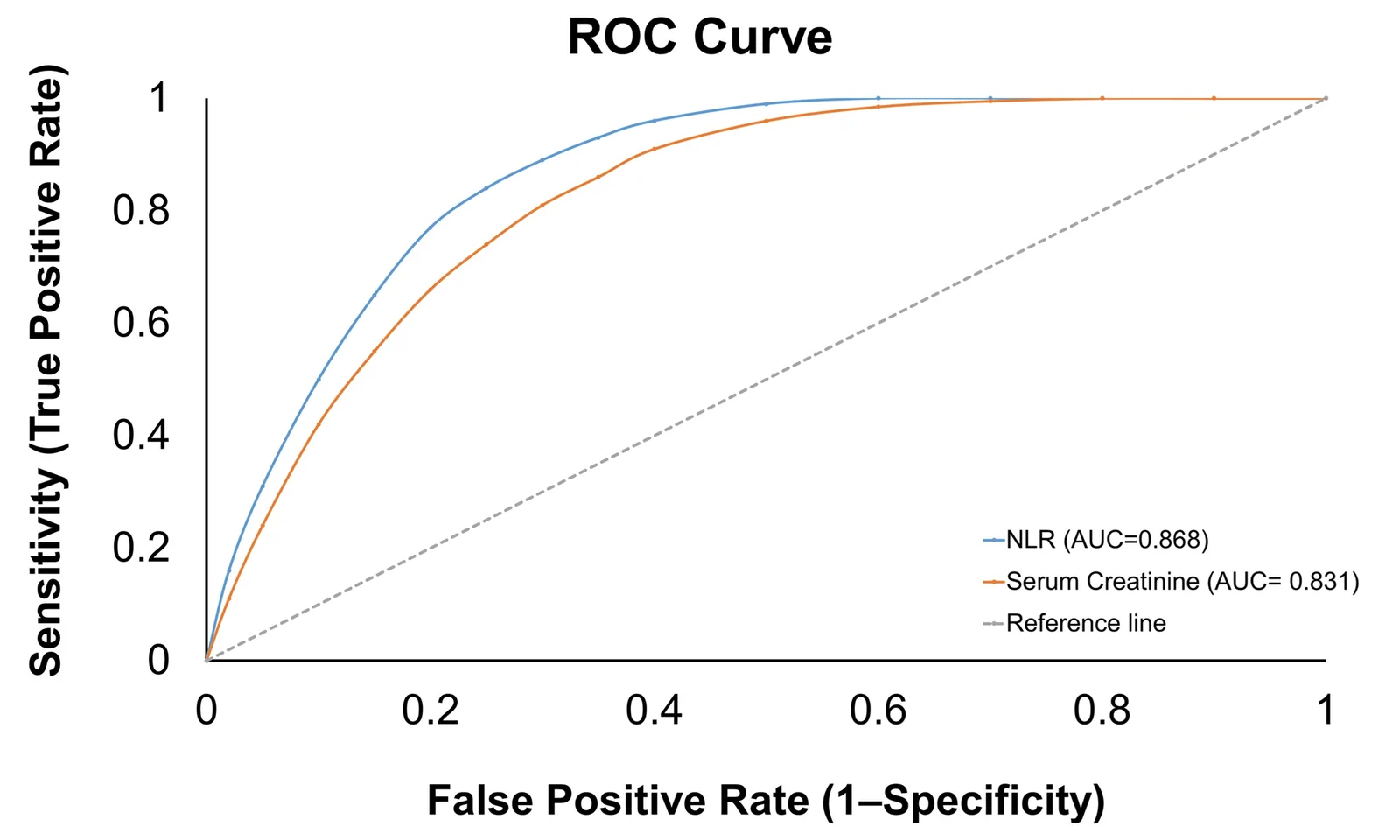
ROC curve of NLR and serum creatinine for prediction of sepsis-related organ dysfunction AUC: area under the curve; NLR: neutrophil-to-lymphocyte ratio; ROC: receiver operating characteristic

ROC curve analysis was performed to assess the predictive ability of NLR and serum creatinine for sepsis-related organ dysfunction. NLR showed good diagnostic performance, with an area under the curve (AUC) of 0.868 (95% CI: 0.831-0.905). At a cutoff value of >8.3, NLR showed 85.2% sensitivity and 79.3% specificity. Serum creatinine also showed good predictive ability, with an AUC of 0.831 (95% CI: 0.790-0.873). At a cutoff value of >1.48 mg/dL, serum creatinine showed 80.3% sensitivity and 75.6% specificity (Table 3). The ROC curve is shown in Figure 1.

**Table 3:** ROC curve analysis for prediction of sepsis-related organ dysfunction ROC curve analysis was used to assess the predictive performance of biomarkers. AUC: area under the curve; NLR: neutrophil-to-lymphocyte ratio; ROC: receiver operating characteristic

Parameter	AUC (95% CI)	Cutoff value	Sensitivity (%)	Specificity (%)	p-value
NLR	0.868 (0.831-0.905)	>8.3	85.2	79.3	<0.001
Serum creatinine	0.831 (0.790-0.873)	>1.48 mg/dL	80.3	75.6	<0.001

Multivariable logistic regression analysis showed that increasing age, diabetes mellitus, chronic kidney disease, NLR, and serum creatinine were independently associated with organ dysfunction (Table 4).

**Table 4:** Multivariable logistic regression analysis for predictors of sepsis-related organ dysfunction Binary logistic regression analysis was used to identify independent predictors of organ dysfunction. NLR: neutrophil-to-lymphocyte ratio

Variable	Adjusted OR	95% CI	p-value
Age (per year increase)	1.03	1.02-1.05	<0.001
Male gender	1.16	0.74-1.82	0.514
Diabetes mellitus	1.92	1.22-3.04	0.005
Hypertension	1.29	0.82-2.04	0.265
Chronic kidney disease	2.58	1.39-4.79	0.003
NLR (per unit increase)	1.28	1.20-1.36	<0.001
Serum creatinine (per mg/dL increase)	3.04	2.06-4.50	<0.001

## Discussion

Sepsis is a serious infection-related condition. It can quickly lead to organ dysfunction if not detected early [14]. In the present study, NLR and serum creatinine showed good value for early detection of sepsis-related organ dysfunction. These tests are simple, low-cost, and easily available in routine hospital laboratories. To our knowledge, this is one of the few studies from Pakistan to evaluate the combined predictive value of NLR and serum creatinine for sepsis-related organ dysfunction. The findings provide locally relevant evidence supporting the use of these inexpensive and routinely available biomarkers for early risk stratification in resource-limited healthcare settings.

Patients with organ dysfunction were older and had more diabetes, hypertension, and chronic kidney disease. This is logical because these patients already have a weak body reserve. In sepsis, infection causes inflammation, poor blood flow, and cellular injury. Older patients and those with chronic diseases are less able to tolerate this stress [15]. Therefore, they are more likely to develop organ dysfunction.

In our study, neutrophil count was higher in patients with organ dysfunction. Neutrophils are the first immune cells that respond to infection. During sepsis, their number increases due to bacterial infection and systemic inflammation. However, excessive neutrophil activity can also damage tissues by releasing enzymes and inflammatory mediators. In addition to neutrophilia, persistent lymphopenia is an important feature of sepsis-induced immune dysregulation. Apoptosis of lymphocytes contributes to immune suppression, reducing the host’s ability to eliminate pathogens and increasing susceptibility to secondary infections. Several studies have associated lymphopenia with prolonged intensive care unit stay, persistent organ dysfunction, and increased mortality, highlighting that the prognostic value of NLR reflects changes in both neutrophil activation and lymphocyte depletion [16]. This may explain the higher neutrophil count in patients with organ dysfunction.

Lymphocyte count was lower in the organ dysfunction group. This finding is also biologically acceptable. Severe sepsis can cause lymphocyte apoptosis and immune suppression. Low lymphocyte count shows poor immune regulation. When neutrophils increase and lymphocytes decrease, the NLR becomes high [17]. Therefore, NLR reflects both inflammation and immune weakness in one simple marker.

The mean NLR was significantly higher in patients with organ dysfunction. ROC analysis also showed good predictive ability of NLR. This finding agrees with previous studies and meta-analyses, which reported that higher NLR is linked with greater sepsis severity, higher SOFA score, and poor outcome [18]. NLR is useful because it is calculated from a routine CBC. It does not need any special kit or expensive test.

Serum creatinine was also significantly higher in patients with organ dysfunction. This is important because the kidney is commonly affected in sepsis. Sepsis can reduce renal perfusion and cause inflammatory injury to kidney tissue [10]. Raised creatinine may show early acute kidney injury or reduced renal clearance [9]. In our study, serum creatinine remained an independent predictor after adjustment for confounders. This supports its role as a practical marker of sepsis-related organ dysfunction.

White blood cell count was also higher in the organ dysfunction group. However, total WBC count alone may be less specific than NLR [7,19]. WBC count can increase due to infection, stress, dehydration, or medications. NLR gives better information because it includes both neutrophil rise and lymphocyte fall [20]. This may be the reason why NLR showed better predictive performance than serum creatinine in our ROC analysis.

Older age, diabetes mellitus, and chronic kidney disease remained independently associated with organ dysfunction after multivariable adjustment. However, these findings should be interpreted cautiously because they are well-established risk factors for adverse outcomes in sepsis, and the cross-sectional design does not permit causal inference. Residual confounding from unmeasured clinical factors may also have influenced these associations [9,21,22]. Nevertheless, NLR and serum creatinine remained significant independent predictors after adjustment, suggesting that both biomarkers provide additional predictive value beyond traditional clinical risk factors. These findings support their potential role in the early risk assessment of septic patients at hospital admission.

Patients presenting with an NLR greater than 8.3 and serum creatinine greater than 1.48 mg/dL may represent a higher-risk subgroup requiring closer clinical observation and more frequent reassessment for the development of organ dysfunction. These readily available biomarkers may complement, but should not replace, established clinical assessment tools. Their use may support early risk stratification and timely escalation of monitoring and supportive care according to the patient’s overall clinical condition. Because both tests are inexpensive and routinely available, they may be particularly useful in resource-limited healthcare settings where access to advanced biomarkers is restricted.

This study has some limitations. First, its single-center, cross-sectional design limits causal inference and may reduce the generalizability of the findings. Second, NLR and serum creatinine were measured only at hospital admission; therefore, temporal changes in these biomarkers and their relationship with disease progression could not be evaluated. Third, disease severity was not adjusted for using standardized severity scores such as SOFA in the multivariable regression model, which may have introduced residual confounding. Finally, external validation in larger multicenter cohorts with serial biomarker measurements is required before these findings can be generalized to broader populations.

## Conclusions

Elevated NLR and serum creatinine were independently associated with sepsis-related organ dysfunction and may serve as simple, inexpensive, and readily available biomarkers for early risk stratification at hospital admission. These findings support their potential use as adjuncts to routine clinical assessment, particularly in resource-limited settings. Future multicenter prospective studies incorporating serial biomarker measurements and standardized severity assessment are needed to validate these findings. Comparative studies evaluating NLR alongside established biomarkers such as procalcitonin may further clarify their relative clinical utility in predicting sepsis outcomes.

## Appendices

Appendix A

**Table 5:** Author-developed data collection pro forma

Section	Variable	Response/recording
Participant information	Study ID	__________
	Date of enrollment	__________
	Age (years)	__________
	Gender	☐ Male ☐ Female
Medical history	Diabetes mellitus	☐ Yes ☐ No
	Hypertension	☐ Yes ☐ No
	Chronic kidney disease	☐ Yes ☐ No
Source of infection	Respiratory infection	☐ Yes ☐ No
	Urinary tract infection	☐ Yes ☐ No
	Abdominal infection	☐ Yes ☐ No
	Other infection	__________________
Laboratory parameters	White blood cell count (× 10⁹/L)	__________
	Neutrophil count (× 10⁹/L)	__________
	Lymphocyte count (× 10⁹/L)	__________
	Neutrophil-to-lymphocyte ratio	__________
	Serum creatinine (mg/dL)	__________
Outcome assessment	Sepsis-related organ dysfunction	☐ Present ☐ Absent
Remarks	Additional clinical notes	_____________________________________________
